# BMI, socioeconomic status, and bone mineral density in U.S. adults: Mediation analysis in the NHANES

**DOI:** 10.3389/fnut.2023.1132234

**Published:** 2023-03-07

**Authors:** Yun Zhang, Caixia Tan, Wenfu Tan

**Affiliations:** Department of Traumatic and Pediatric Orthopedics, The Second Affiliated Hospital, Hengyang Medical School, University of South China, Hengyang, China

**Keywords:** bone mineral density, mediation effect, NHANES, socioeconomic status, body mass index

## Abstract

**Introduction:**

The mechanism by which socioeconomic status (SES) affects bone mineral density (BMD) remains unknown, and body mass index (BMI) may be a potential mediator. The purpose of this study was to investigate whether BMI mediates the relationship between SES [education level and poverty income ratio (PIR)] and lumbar BMD and the proportion it mediates.

**Methods:**

This study included a total of 11,075 adults from the National Health and Nutrition Examination Survey (NHANES). Lumbar BMD was measured at the lumbar spine by dual-energy X-ray absorptiometry (DXA). Multivariate linear regression and smoothing curve fitting were used to investigate the relationship between SES and lumbar BMD. Mediator analysis was used to investigate the proportion of BMI mediating the association between SES and BMD.

**Results:**

In the fully adjusted model, there was a positive correlation between SES and BMD (education level: β = 0.025, 95% CI: 0.005, 0.045; PIR: β = 0.007, 95% CI: 0.002, 0.011). Mediation analysis showed that BMI mediated the relationship between PIR, education level, and lumbar BMD with a range of mediation proportions from 13.33 to 18.20%.

**Conclusion:**

BMI partially mediated the positive association between SES and BMD, and this association may be largely mediated by factors other than BMI.

## 1. Introduction

Osteoporosis is a bone disease characterized by impaired bone strength that puts individuals at increased risk of fractures in the spine and joint areas (1, 2). As the global population ages, osteoporosis imposes a heavy socioeconomic and public health burden (3). The annual cost of osteoporosis fracture prevention and treatment in the United States is expected to exceed $50 billion 20 years from now (4, 5).

Investigation of risk factors for osteoporosis is an important tool for maintaining bone mass and reducing fracture risk (6). In addition to common laboratory and screening indicators (e.g., blood lipids, body composition, etc.) (7, 8), sociological factors are receiving increasing attention in bone metabolism (9). Wang and Dixon used multiple linear regression to investigate and find a significant positive association between education level, poverty income ratio (PIR), and BMD in menopausal women (10). A recent cross-sectional study in adult men again validated this association and highlighted the importance of socioeconomic status (SES) in the management of osteoporosis (11). However, the mechanisms behind the association between SES and BMD are complex and unclarified. Available evidence suggests that this association may arise primarily from the indirect effects of potential mediators, and exploring the main mediators is important for targeting groups with unequal SES for the prevention and management of osteoporosis (12, 13).

Individuals with low SES are often associated with problems such as inadequate energy intake (14) and lack of essential nutrients (15), which may lead to an unhealthy body mass index (BMI) or waist circumference. On the other hand, BMI has long been considered to be strongly associated with SES as a protective factor against bone loss (16, 17). Given these associations, BMI is considered to be a potentially important factor in mediating the relationship between SES and BMD.

Therefore, a cross-sectional study based on the four cycles of National Health and Nutrition Examination Survey (NHANES) 2011–2018 was carried out, to investigate the mediating role of BMI in the association between SES and lumbar BMD.

## 2. Materials and methods

### 2.1. Study population and data source

**FIGURE 1:**
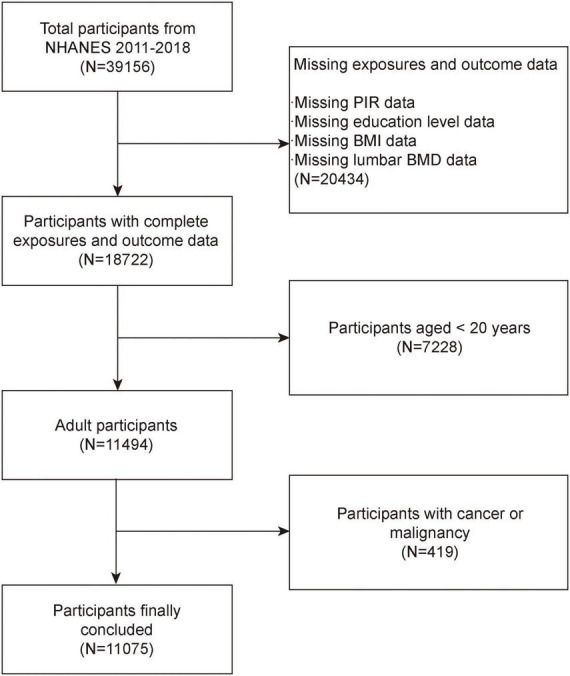
Flow chart of participants selection. NHANES, National Health and Nutrition Examination Survey; BMD, bone mineral density; BMI, body mass index; PIR, poverty income ratio.

The NHANES is a comprehensive, national survey that collects health and nutrition information from non-institutionalized civilian residents in the United States (18, 19). The National Center for Health Statistics (NCHS) Research Ethics Review Board authorized the study protocol. At the time of recruiting, all subjects provided written consent. According to inclusion exclusion criteria, excluded 20,434 participants without SES data or BMD data, 7,228 participants age less than 20 years and 419 samples with cancer or malignancy. The study eventually included 11,075 participants (Figure 1).

### 2.2. Study variables

The exposure variable is SES, which consists of PIR and educational attainment. PIR is a continuous variable, which is the rate of self-reported household income, based on household or family size, household age composition and year. Educational level is a categorical variable and is divided into three groups less than high school, high school, and more than high school. BMI was calculated according to international standards: weight divided by height squared. Outliers will receive reasonable verification to ensure the credibility of the data. For BMI classification according to WHO standards (underweight <18.5, normal 18.5–24.9, overweight 25–29.9, obese ≥30 kg/m^2^). Lumbar BMD was measured as the primary outcome of this study by dual-energy X-ray absorptiometry. Age, gender, race, diabetes status, smoke status, high blood pressure status, total calcium, serum phosphorus, blood urea nitrogen, activities status, direct HDL cholesterol, serum creatinine, and total cholesterol were all covariates in this study. The interpretation, measurement and calculation of all variables can be found on the official NHANES website.^1^

### 2.3. Statistical analysis

All analyses were performed with R (version 4.2) and Empowerstats (version 4.1). The Chi-square test and *t*-test were used to assess the demographic characteristics of the participants by BMI subgroups. Multivariate logistic regression analyses were used to investigate the association between SES, BMI and lumbar BMD (20–22). The potential mediated effect of BMI on the association between SES and lumbar BMD was estimated by parallel mediator analysis. The parallel mediation model uses individual indicators as mediators. The direct effect (DE) is the effect of SES on lumbar BMD without mediators. Indirect effects (IE) are the consequences of SES on lumbar BMD that are mediated by mediators. The fraction of mediators was estimated by dividing IE by TE (total effect).

## 3. Results

### 3.1. Baseline characteristics

Table 1 shows the weighted characteristics of the participants stratified by BMI. A total of 5,717 male and 5,358 female adults participated, of whom 190 were underweight (1.72%), 3,235 were normal (29.21%), 3,465 were overweight (31.29%), and 4,185 were obese (37.77%). All variables except smoking status differed significantly (*P* < 0.05) at baseline characteristics according to BMI category. Underweight and obese participants tended to have lower income and education and lower lumbar BMD compared to normal weight and overweight participants.

**TABLE 1:** Characteristics of the participants.

Outcome	BMI (kg/m^2^) categorical				*P*-value
	**Underweight <18.5 (*N* = 190)**	**Normal 18.5–24.9 (*N* = 3,235)**	**Overweight 25.0–29.9 (*N* = 3,465)**	**Obese ≥30.0 (*N* = 4,185)**	
Age (years)	30.873 ± 12.208	36.430 ± 11.981	40.156 ± 11.254	40.382 ± 11.266	<0.001
Gender (%)					<0.001
Male	41.352	47.037	60.234	51.013	
Female	58.648	52.963	39.766	48.987	
Race (%)					<0.001
Non-Hispanic White	57.877	63.817	60.201	57.377	
Non-Hispanic Black	15.167	9.699	10.492	15.500	
Mexican American	3.713	6.092	11.468	13.044	
Other race	23.243	20.392	17.839	14.079	
PIR	2.071 ± 1.402	2.970 ± 1.703	3.068 ± 1.675	2.825 ± 1.635	<0.001
Education level (%)					<0.001
Less than high school	13.520	11.734	14.293	14.017	
High school	26.111	19.317	21.358	24.438	
More than high school	60.369	68.949	64.349	61.545	
Moderate activities (%)					<0.001
Yes	35.733	29.998	30.966	29.813	
No	64.267	70.002	69.034	70.187	
Diabetes status (%)					<0.001
Yes	1.280	2.172	4.247	9.543	
No	98.720	97.828	95.753	90.457	
High blood pressure status (%)					<0.001
Yes	6.458	10.681	20.258	33.173	
No	93.542	89.319	79.742	66.827	
Smoking status (%)					0.359
Ever	39.620	38.337	41.193	41.637	
Never	60.380	61.663	58.807	58.363	
Total calcium (mmol/L)	2.361 ± 0.080	2.352 ± 0.081	2.346 ± 0.084	2.331 ± 0.084	<0.001
Total cholesterol (mmol/L)	4.457 ± 0.787	4.771 ± 0.983	5.070 ± 1.034	5.045 ± 1.041	<0.001
Direct HDL cholesterol (mmol/L)	1.572 ± 0.410	1.556 ± 0.439	1.340 ± 0.374	1.218 ± 0.332	<0.001
Serum phosphorus (mmol/L)	1.260 ± 0.178	1.223 ± 0.176	1.192 ± 0.178	1.183 ± 0.180	<0.001
Blood urea nitrogen (mmol/L)	4.174 ± 1.841	4.459 ± 1.461	4.709 ± 1.493	4.576 ± 1.578	<0.001
Creatinine (mmol/L)	70.300 ± 35.257	74.160 ± 22.315	77.813 ± 20.627	75.777 ± 27.316	<0.001
Lumbar BMD (g/cm^2^)	0.983 ± 0.125	1.044 ± 0.146	1.044 ± 0.146	1.036 ± 0.154	<0.001

Mean + SD for continuous variables: *P*-value was calculated by weighted linear regression model. % For categorical variables: *P*-value was calculated by weighted Chi-square test. BMD, bone mineral density; PIR, the ratio of family income to poverty; BMI, body mass index.

### 3.2. Association between SES and BMI with lumbar BMD

Table 2 shows the results of multivariate logistic regression analysis with a positive association between SES and lumbar BMD. There was a significant positive linear association between PIR and lumbar BMD, with an increase in lumbar BMD of 0.007 g/cm^2^ per unit increase in PIR (β = 0.007, 95% CI: 0.002, 0.011). This association also existed between education level and lumbar BMD, participants with more than high school education having a 0.025 g/cm^2^ higher lumbar BMD than those with less than high school education (β = 0.025, 95% CI: 0.005, 0.045). And participants with high school education having a 0.019 g/cm^2^ higher lumbar BMD than those with less than high school education (β = 0.019, 95% CI: 0.003, 0.035).

**TABLE 2:** Association between SES and BMI with lumbar BMD (g/cm^2^).

Subgroups	Model 1 [β (95*%* CI)]	Model 2 [β (95*%* CI)]	Model 3 [β (95*%* CI)]
PIR	0.006 (0.001, 0.011)	0.005 (0.001, 0.009)	0.007 (0.002, 0.011)
**Education level**			
Less than high school	Reference	Reference	Reference
High school	0.017 (0.007, 0.027)	0.019 (0.006, 0.032)	0.019 (0.003, 0.035)
More than high school	0.030 (0.021, 0.039)	0.027 (0.009, 0.045)	0.025 (0.005, 0.045)
Body mass index (kg/m^2^)	0.001 (0.000, 0.001)	0.001 (0.000, 0.001)	0.001 (0.001, 0.002)
**Categories**			
Underweight (<18.5)	Reference	Reference	Reference
Normal (18.5–24.9)	0.006 (0.003, 0.009)	0.009 (0.006, 0.012)	0.012 (0.007, 0.017)
Overweight (25.0–29.9)	–0.001 (–0.004, 0.002)	0.000 (–0.003, 0.004)	0.002 (–0.004, 0.008)
Obese (≥30)	0.003 (0.003, 0.004)	0.003 (0.002, 0.004)	0.003 (0.001, 0.004)
*P* for trend	0.094	0.225	0.609
**Subgroup analysis stratified by gender**			
Males	0.001 (0.000, 0.002)	0.001 (0.001, 0.002)	0.002 (0.001, 0.004)
Females	0.001 (0.000, 0.001)	0.001 (0.000, 0.001)	0.001 (–0.000, 0.002)

Model 1: no covariates were adjusted. Model 2: age, gender, and race were adjusted. Model 3: age, gender, race, BMI, activities status, diabetes status, smoke status, high blood pressure status, total calcium, total cholesterol, direct HDL cholesterol, blood urea nitrogen, serum creatinine, and serum phosphorus were adjusted. In the subgroup analysis stratified by gender, the model is not adjusted for the stratification variable itself; in model 3, BMI was not adjusted for in the association between the continuous and categorical variables of BMI and BMD. BMD, bone mineral density; PIR, the ratio of family income to poverty; SES, socioeconomic status.

The results of the multiple logistic regression analysis showed a positive relationship between BMI and lumbar BMD, and this association remained significant and stable in all models (Table 2). For every 1 kg/m^2^ increase in BMI, lumbar BMD increased by 0.001 g/cm^2^ (β = 0.001, 95% CI: 0.001, 0.002). In contrast, when BMI was transformed into a categorical variable for analysis, this relationship became reversed and insignificant in overweight participants. When subgroup analysis was performed by gender, the relationship between BMI and lumbar BMD showed a positive association in both male and female participants.

**FIGURE 2:**
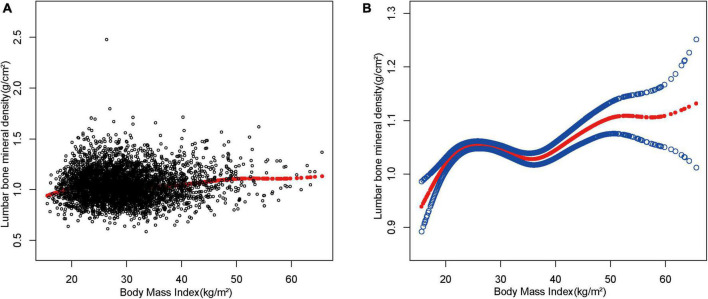
The association between body mass index and lumbar bone mineral density. **(A)** Each black point represents a sample. **(B)** The solid red line represents the smooth curve fit between variables. Blue bands represent the 95% of confidence interval from the fit.

Considering that the results were not significant in the sensitivity analysis, smoothed curve fitting was further utilized to confirm the non-linear relationship between BMI and lumbar spine BMD. The results showed a non-linear positive relationship between BMI and lumbar BMD with saturated values (Figure 2).

### 3.3. Mediation analysis

**FIGURE 3:**
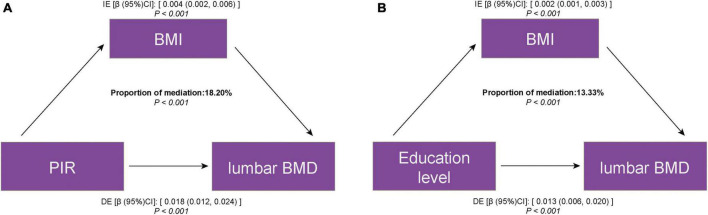
Estimated proportion of the association between SES and lumbar BMD mediated by BMI. **(A)** PIR and lumbar BMD; **(B)** education level and lumbar BMD. IE, indirect effect; DE, direct effect; mediation proportion = IE/DE + IE.

The mediation analysis investigated whether and to what extent BMI mediated the association between SES and lumbar BMD. Table 3 shows the total effect, which is the effect of SES on lumbar BMD; the direct effect, which is the effect of PIR, education level on lumbar BMD, not mediated by BMI; and the indirect effect, which is the effect of PIR, education level on lumbar BMD, mediated by BMI. In general, the direct effect greatly exceeded the indirect effect, although the statistical significance of the latter was significant. The proportion of BMI mediating the effect of PIR and education level on lumbar BMD was 18.20 and 13.33%, respectively (Figure 3).

**TABLE 3:** Body mass index as a mediator in the associations of SES with lumbar BMD (g/cm^2^).

Mediation effect (SES – BMI – lumbar BMD)	PIR [β (95%) CI]	Education level [β (95%) CI]
Total effect	0.022 (0.020, 0.034)	0.015 (0.17, 0.028)
Direct effect	0.018 (0.012, 0.024)	0.013 (0.006, 0.020)
Indirect effect	0.004 (0.002, 0.006)	0.002 (0.001, 0.003)
Mediated (%)	18.20	13.33

Model was adjusted for age, gender, race, activities status, diabetes status, smoke status, high blood pressure status, total calcium, total cholesterol, direct HDL cholesterol, blood urea nitrogen, serum creatinine, and serum phosphorus. IE, indirect effect; DE, direct effect; mediation proportion = IE/DE + IE. BMD, bone mineral density; BMI, body mass index; PIR, the ratio of family income to poverty; SES, socioeconomic status.

## 4. Discussion

In the present study, the results of the multiple regression analysis suggest that US adults with higher SES are associated with higher lumbar BMD. More importantly, this study found that BMI mediated the positive association between SES and lumbar BMD, although the proportion of mediation was less than 20%. This suggests that the association between SES and BMD may be primarily due to factors other than BMI, such as genetics, dietary intake, and levels of systemic inflammation.

For most causes of morbidity and mortality, SES is of great importance and impact (23–25). Therefore, the study of SES as a risk factor for bone health is essential to reduce the public health burden. The association between SES and BMD has been of interest to researchers for 30 years, but results have been inconsistent due to differences in populations and study methods (26). Fehily et al. in 1992 investigated factors that may have influenced BMD during the development of over 500 14 years-olds and showed that males in manual occupations may have higher BMD (27). Results from the Louisiana Osteoporosis Study suggest a positive association between SES and BMD in the total population, with males with lower education and females with lower income being the most susceptible to relatively lower BMD (28). A meta-analysis including eight epidemiological studies showed that most population-based studies support the idea that participants with higher income levels and education are more likely to have higher BMD (29). This finding was also validated in this cross-sectional study, which included 11,075 representative US participants. However, the reasons behind the positive association between SES and BMD are complex and unexplained. Based on the available evidence, the negative effects of SES on BMD are thought to possibly stem from unhealthy lifestyles, including factors such as food insecurity (30), lack of essential nutrients (31, 32), and exposure to harmful substances (33). Health outcomes of an unhealthy lifestyle, such as underweight (34) and visceral fat accumulation (35, 36), may further negatively affect bone metabolism.

In the past, obesity and being overweight have been considered a protective factor. A positive association between BMI and BMD was found in several studies as early as 20 years ago (37, 38). Researchers concluded that BMI reduced the risk of bone loss and fracture in gender-specific populations and groups of menopausal women (39, 40). The results of multivariate logistic regression and subgroup analyses also indicated a positive association between BMI and BMD, which was maintained significantly in both men and women. The mechanisms underlying the positive association between obesity and BMD have long been described. The main include: (1) the mechanical overload generated in the presence of obesity leads to bone deformation, which triggers a series of transduction signals that stimulate increased bone mass through increased osteoblast activity; (2) increased osteogenic differentiation and osteoblast maturation of mesenchymal stem cells through adipocyte production of sex steroids; and (3) adipose tissue is a substrate for sex hormone synthesis and secretes adipokines and cytokines, which play a role in bone metabolism. Given these mechanisms, obesity is thought to be a potentially important mediator of the association between SES and BMD, and the results of the mediation analysis support this hypothesis.

Exploring the main mediators of the relationship between SES and BMD is important for the prevention and management of osteoporosis (11). The data suggest that the effects of SES on BMD are broad and complex and may affect bone metabolism in a variety of ways, including through diet, inflammation, and physical activity patterns (32, 41–43). However, a significant proportion of these can have a large effect on body size, with changes in both diet and physical activity patterns leading to corresponding changes in BMI, which can further influence bone metabolic processes (44). The results of mediating effects analysis suggest that BMI is indeed a mediator of the relationship between SES and BMD, but the proportion of mediators for both PIR and education level is below 20%, implying that there may be other major mediators. Dietary intake factors may be worth investigating. Lim et al. investigated calcium intake among adults in six regions of Korea, and the authors found significant regional differences in calcium intake. Furthermore, participants with lower SES had inadequate calcium intake and low diet quality, and inadequate calcium and energy intake may have a negative impact on bone metabolism (45). In addition, inflammation levels may also be an important factor in the association between SES and BMD (46). It has been shown that lower SES is associated with increased psychosocial stress and elevated blood inflammation levels, and higher levels of systemic inflammation have been shown to be negatively associated with BMD in menopausal women (47). In addition, higher dietary inflammatory potential has also been suggested as a risk factor for bone health, and a meta-analysis that included more than 100,000 participants suggested that a diet high in pro-inflammatory components may increase the risk of osteoporosis and fracture (48).

Our study has some limitations. First, due to the design of the cross-sectional study, the current study were unable to determine the causal relationship between SES and lumbar BMD. In addition, self-reported SES may lead to data bias and affect the accuracy of conclusions (49, 50). Despite these shortcomings, our study has several advantages. This study includes data from a large and representative cross-sectional survey. More importantly, this study confirms the association between SES and lumbar BMD and extends these studies for the first time to the potential mediation effects of BMI.

## 5. Conclusion

According to the findings of this study, BMI partially mediates the positive relationship between SES and BMD. Further investigation is needed to determine whether there are higher mediating variables than BMI in this association, such as dietary intake and inflammation levels.

## Data availability statement

Publicly available datasets were analyzed in this study. This data can be found here: https://wwwn.cdc.gov/nchs/nhanes/ContinuousNhanes.

## Ethics statement

The studies involving human participants were reviewed and approved by the NCHS Ethics Review Board. The patients/participants provided their written informed consent to participate in this study.

## Author contributions

YZ and WT designed the research and revised the manuscript. YZ and CT collected and analyzed the data and drafted the manuscript. All authors contributed to the manuscript and approved the submitted version.

## Footnotes

1
https://www.cdc.gov/nchs/nhanes/
